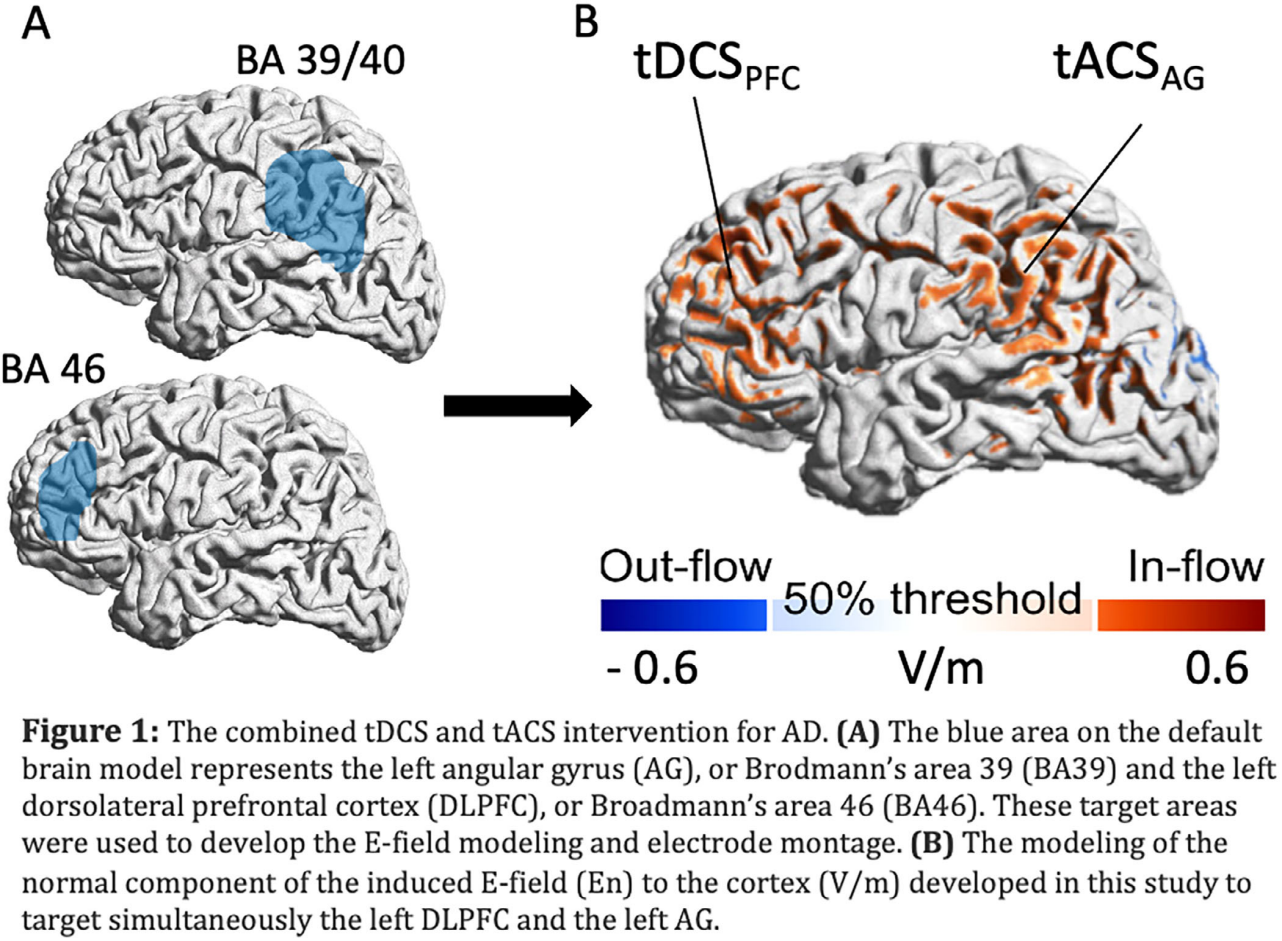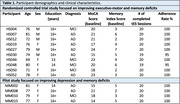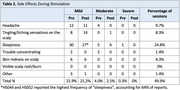# Home‐Based Multifocal Brain Circuit‐Based Neuromodulation Combining tACS and tDCS in Older Adults with Alzheimer’s Disease

**DOI:** 10.1002/alz.089723

**Published:** 2025-01-09

**Authors:** Talia Gilfix, Judy Zheng, Jonathan Davis, Rachel Fox, Benjamin Stein, Wanting Yu, Jasmine Morgan, Alisha Syed, Maggie L Syme, Alexander Opitz, Bradley Manor, Alvaro Pascual‐Leone, Davide Cappon

**Affiliations:** ^1^ Hinda and Arthur Marcus Institute for Aging Research at Hebrew SeniorLife, Boston, MA USA; ^2^ Deanna and Sidney Wolk Center for Memory Health at Hebrew SeniorLife, Boston, MA USA; ^3^ University of Minnesota, Minneapolis, MN USA; ^4^ Harvard Medical School, Boston, MA USA

## Abstract

**Background:**

Alzheimer’s disease (AD) affects over 55 million people worldwide and is characterized by abnormal deposition of amyloid‐β and tau in the brain causing neuronal damage and disrupting transmission within brain circuits. Episodic memory loss, executive deficits, and depression are common symptoms arising from altered function in spatially distinct brain circuits that greatly contribute to disability. Transcranial electrical stimulation (tES) can target these circuits and has shown promise to relieve specific symptoms. However, previous trials focused on a single symptom and have been limited by poor quantification of induced electric fields (E‐field) in the intended cortical target(s). The studies aim to provide multi‐symptom relief to older adults with AD by combining two types of tES.

**Method:**

Fourteen participants diagnosed with mild cognitive impairment (MCI) or early dementia due to AD were recruited as part of two studies (Table 1). Optimization of tES was performed by modeling the normal component of the induced E‐field (En) to target the left dorsolateral prefrontal cortex (DLPFC, Brodmann area 46) with transcranial direct current stimulation (tDCS) and to target the left angular gyrus (AG, Brodmann areas 39/40) with transcranial alternating current stimulation (tACS – 40 Hz) (Figure 1). Participants received daily stimulation sessions for four weeks at home, with baseline, post‐intervention, and 3‐month follow‐up assessments. E‐field modeling using MRIs evaluates behavioral effects' dependency on E‐field induced by tDCS and tACS in DLPFC and AG.

**Result:**

Both studies showed excellent adherence to a home‐based, multi‐symptom tDCS/tACS intervention. To date, out of 280 scheduled sessions across 14 participants, 278 were completed, with a 99% adherence rate. The most common side effects were mild and transient (Table 2). Structural MRI scans were used to quantify E‐field modeling in target brain regions.

**Conclusion:**

The studies demonstrate the safety, feasibility, and adherence of a remote‐supervised, caregiver‐led home‐based intervention combining tDCS and tACS to target two distinct brain networks and thus induce a more meaningful clinical impact by reducing distinct disabilities in AD. Quantifying the induced E‐field will provide data to assess the mediating effects of E‐field on treatment outcomes, yielding critical insights to enable future larger‐scale trials.